# Low‐dose ASA therapy does not alter core or skin temperature during hot‐dry or warm‐humid heat stress (PSU HEAT project)

**DOI:** 10.14814/phy2.70375

**Published:** 2025-05-11

**Authors:** Kat G. Fisher, Olivia K. Leach, Rachel M. Cottle, Lacy M. Alexander, W. Larry Kenney

**Affiliations:** ^1^ Center for Healthy Aging Pennsylvania State University University Park Pennsylvania USA; ^2^ Department of Kinesiology Pennsylvania State University University Park Pennsylvania USA; ^3^ Graduate Program in Physiology Pennsylvania State University University Park Pennsylvania USA

**Keywords:** aging, aspirin, cardiovascular health, heat stress, skin blood flow, thermoregulation

## Abstract

Nearly 40% of US adults over the age of 50 use aspirin (ASA) therapy for the primary or secondary prevention of cardiovascular disease. Systemic platelet cyclooxygenase inhibition with low‐dose ASA attenuates reflex cutaneous vasodilation and accelerates the rate of rise of core temperature during passive heating in middle‐aged adults. The functional effect of low‐dose ASA therapy on thermoregulatory and cardiovascular responses to hot and humid environmental extremes in older (>65 years) adults has not been determined. Eleven older adults (5F; 66–80 years) were exposed to progressive heat stress in an environmental chamber at a metabolic rate comparable to activities of daily living (~80 W∙m^−2^) in a warm‐humid (WH; 36°C, 52% rh) and hot‐dry (HD; 40°C, 21% rh) environment following 7 days of low‐dose ASA (81 mg/day) or placebo. Core temperature (T_c_), skin temperature (T_sk_), heart rate (HR), mean arterial pressure (MAP) and forearm blood flow (FBF) were measured, and rate‐pressure product was subsequently calculated. Low‐dose ASA attenuated FBF and forearm vascular conductance (all *p* ≤ 0.04) but had no effect on T_c_ or T_sk_ in either environment. In conclusion, low‐dose ASA attenuates the skin blood flow response during minimal activity heat stress in both dry and humid environments but does not alter T_c_.

## 1 INTRODUCTION

Low‐dose aspirin (ASA) therapy has historically been and is still widely used for cardiovascular disease (CVD) prevention, with nearly 30 million adults aged 40 or older taking daily baby ASA for primary prevention of CVD (O'Brien et al., 2019). Low doses of ASA can protect from platelet‐mediated thrombotic events (Patrono et al., 2001). Through the first pass effect, low‐dose ASA is an irreversible inhibitor of cyclooxygenase (COX) and subsequently decreases platelet aggregation and vasoconstriction through reduced thromboxane A_2_ (TXA_2_). However, high‐dose ASA can also inhibit vascular COX and subsequent production of prostacyclin (PGI_2_), a platelet aggregation inhibitor and vasodilator (Patrono et al., 2001). Depending on the dosage, ASA therapy can alter both platelet aggregation and COX‐mediated vascular signaling (Pedersen & FitzGerald, 1984).

Previously, our lab found that in otherwise healthy middle‐aged adults (57 ± 3 years), low‐dose ASA therapy attenuated reflex cutaneous vasodilation (Holowatz & Kenney, 2009) and reduced the time to increase oral temperature by 1.0°C during passive heating in a water‐perfused suit (Holowatz et al., 2010). However, reflex cutaneous vasodilation was unaltered when vascular COX was inhibited locally through intradermal microdialysis administration of a non‐specific COX inhibitor (Holowatz et al., 2009). Low‐dose ASA therapy prevents acetylation of platelet COX‐1 and inhibits COX for the life of the platelet, potentially preventing activation of platelets and the subsequent release of factors that stimulate signaling pathways involved in cutaneous reflex vasodilation (Förstermann et al., 1988; Kaul et al., 1992; Oskarsson & Hofmeyer, 1996). This suggests that ASA administered systemically alters reflex cutaneous vasodilation primarily through COX‐1 mediated platelet mechanisms.

Subsequently, our lab examined the functional consequences of low‐dose ASA therapy to heat stress at rest and during exercise at 60%VO_2max_ in an environmental chamber (30°C, 40% relative humidity) in a cohort of middle‐aged adults (50–65 years) (Bruning et al., 2013). Following 40 min of passive heating, core temperature (T_c_) was significantly elevated in the ASA condition, and that elevation was sustained throughout exercise. Additionally, the onset of reflex cutaneous vasodilation was shifted to higher mean body temperatures in the ASA condition.

Therefore, the primary purpose of the present study was to examine the effect of 7 days of low‐dose (81 mg/day) ASA therapy in older adults (>65 years) on T_c_ and cardiovascular responses during dry and humid heat stress during minimal activities of daily living. In order to maximize the chances of finding an effect when convective heat loss would be altered and to characterize the impact of ASA at dry and humid environmental extremes, progressive heating was used until hot‐dry (HD; 40°C and 21%) and warm‐humid (WH; 36°C and 52% rh) environments were reached that were the same for all subjects. We hypothesized that ASA therapy would result in elevated T_c_ and attenuated skin blood flow in the warm‐humid and hot‐dry environments.

## 2 METHODS

### 2.1 Subjects

All testing was conducted in controllable environmental chambers housed in Noll Laboratory at the Pennsylvania State University, and all procedures were approved by the university's Institutional Review Board and conformed to the Declaration of Helsinki's stated guidelines. Participants gave oral and written consent after being informed of all aspects of the experimental study. All experimental procedures are registered on ClinicalTrials.gov (NCT0428439).

Subject characteristics are presented in Table 1. Subjects were representative of the population in these age groups with respect to body size and aerobic fitness (Kaminsky et al., 2015; Liguori, 2020). Subjects refrained from taking ASA, anti‐thrombotic therapy, or other anti‐inflammatory therapies for at least 2 weeks prior to participating in the study. Maximal aerobic capacity (V̇O_2max_) was determined using open‐circuit spirometry (Parvo Medics TrueOne® 2400, Parvo, UT, USA) during a graded exercise test performed on a motor‐driven treadmill. During the experiments, subjects wore thin, short‐sleeved cotton tee‐shirts, shorts, socks, and walking/running shoes plus sports bras for the women.

**TABLE 1:** Subject characteristics.

*n*	11 (5 females)
Age (year)	71 (66–80)
Height (m)	1.7 ± 0.1
Weight (kg)	73.3 ± 13.2
BMI (kg∙m^−2^)	25.3 ± 3.5
A_D_ (m^2^)	1.84 ± 0.19
A_D_∙mass^−1^ (m^2^∙kg^−1^)	0.025 ± 0.002
VO_2max_ (ml∙kg^−1^∙min^−1^)	29.7 ± 11.6

Abbreviations: A_D_, DuBois body surface area; A_D_·mass^−1^, body surface area‐to‐mass ratio; Vo_2max_, maximal oxygen consumption.

### 2.2 Blinded drug treatments

Nonidentifiable capsules were compounded by a registered pharmacist and given to subjects to take once daily for 7 days prior to experimental trials. Subjects continued the treatment until completion of experimental trials for a maximum of 14 days of treatment. The study design was a randomized, double‐blind, placebo‐controlled crossover study with 81 mg of ASA (Bayer) or placebo. The duration and dose of ASA were chosen because ASA has shown to fully inhibit platelet aggregation within 4–6 days (Patrono et al., 2001). As an exploratory follow‐up to rule out the potential of vascular COX‐inhibition, a subset (4 subjects) of participants completed experimental trials after a single maximum recommended dose (650 mg) of ASA. Neither subjects nor investigators were blinded to this trial. Subjects were instructed to take experimental medication 1 h. Prior to the experiment. This timeframe was chosen because the pharmacokinetics and dynamics of oral ASA reach peak plasma concentrations in 1 h (Rocca & Petrucci, 2012). A minimum of a 2‐week washout period separated experimental trials for a given experimental medication regimen.

### 2.3 Experimental procedures

Before each experimental session, subjects were instructed to abstain from alcoholic beverages and vigorous exercise for 24 h and from caffeine for 12 h. All participants were euhydrated, as defined as urine specific gravity ≤1.020 (USG; PAL‐S, Atago, Bellevue, WA) prior to the start of the experiment (Kenefick & Cheuvront, 2012). Subjects performed light physical activity in an environmental chamber at a low metabolic intensity reflecting the metabolic demand of activities of daily living (net metabolic heat production of ~83 W·m^−2^). This metabolic rate was chosen given that older adults are likely to continue to perform activities of daily living during a heat wave, but unlikely to engage in more vigorous physical activity. Subjects cycled on a cycle ergometer (Lode Excalibur, Groningen, The Netherlands) against zero resistance at a cadence of 40–50 rpm.

Subjects cycled continuously for 120 min during a progressive heat stress protocol in a controllable environmental chamber. Either (1) water‐vapor pressure (P_a_) was held constant at 12 mmHg and dry‐bulb temperature (T_db_) was increased in a stepwise fashion of 1°C every 5 min following a 30 min equilibration period (hot‐dry condition) or (2) T_db_ was held constant at 36°C and P_a_ was continuously increased in a stepwise fashion of 1 mmHg every 5 min following a 30 min equilibration period (warm‐humid condition). The progressive heating or humidification protocols identified 2 common conditions attained by all participants: 40°C and 21% rh in the hot‐dry condition or 36°C and 52% rh in the warm‐humid condition. While all participants underwent a 2 h. exposure, these environments occurred at different time points of the exposure due to different starting points. Therefore, the data collected at 40°C and 21% rh in the hot‐dry condition or 36°C and 52% rh in the warm‐humid condition ranged from minute 30 to minute 90 of the exposure. Outcome variables were compared between trials ASA and placebo trials within a subject.

### 2.4 Measurements

Continuous measurements of T_c_ were recorded using gastrointestinal temperature telemetry capsules (VitalSense, Philips Respironics, Bend, OR, USA; BodyCap, Hérouville‐Saint‐Clair, France). Subjects ingested the capsules 1–2 h before reporting to the laboratory in accordance with previously published data demonstrating that ingestion times from 1 to 12 h before use do not influence the precision of T_c_ data (Notley et al., 2021). Skin temperature was measured continuously on each subject's chest (T_chest_), arm (T_arm_), thigh (T_thigh_), and lower leg (T_leg_) and was used to calculate a weighted mean skin temperature (T_sk_) (Ramanathan, 1964):
(1)

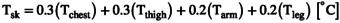




The rate of oxygen uptake (VO_2_) and respiratory exchange ratio (RER) were measured twice during experimental trials at 5 and 60 min using open‐circuit spirometry (Parvo Medics TrueOne® 2400, Parvo, UT, USA). Average VO_2_ and RER values from the two time points (which did not differ significantly) were used to calculate metabolic rate. Metabolic rate [M; Watts (W)], normalized to body surface area, was calculated from V̇o_2_ and RER (Cramer & Jay, 2019) as:
(2)

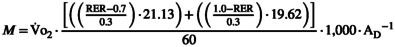

where A_D_ is the Dubois surface area (m^2^). Because minimal external work (W) was done by participants, M equaled net metabolic rate (M_net_).

Heart rate (HR) was continuously measured, and mean arterial pressure (MAP) was measured at the beginning and end of the equilibration period and every 5 min thereafter throughout the experimental trial. Blood pressure was taken using an automated blood pressure monitor (CardioCap; GE Healthcare, Milwaukee, WI). Rate pressure product (RPP) was calculated by multiplying HR by systolic blood pressure.

Forearm blood flow (FBF) was measured at the beginning and end of the equilibration period and every 5 min thereafter throughout the experimental trial by venous occlusion plethysmography using a mercury in silastic strain gauge (EC6 Plethysmograph; Hokanson, Bellevue, WA) while blood flow to the hand was occluded (Whitney, 1953). Data were collected using Powerlab and Labchart software (ADInstruments, Colorado Springs, CO). FBF was calculated in accordance with standardized procedures (Whitney, 1953). Forearm vascular conductance (FVC) was calculated as FBF/MAP.

In order to determine the efficacy of the ASA treatment, platelet aggregation properties were analyzed. Blood samples were drawn immediately prior to entering the environmental chamber and upon completion of the 120 min progressive heat stress protocol. Whole blood samples were collected in 2.7 mL sodium citrate‐lined blood sample tubes (9:1 ratio, BD Vacutainer®, Franklin Lakes, NJ, USA). Platelet aggregation properties were analyzed in both pre‐test and post‐test blood samples within 3 h following blood draw using whole blood impedance aggregometry via CHRONO‐LOG® Model 700 Whole Blood/Optical Lumi‐Aggregometer and associated AGGRO/LINK®8 software (Chrono‐log Corporation, Havertown, PA) previously described in detail (Williams et al., 2024). To induce platelet aggregation, 10 μL of 50 mM arachidonic acid (Chrono‐log Corporation, Havertown, PA) was added to the blood sample. Following addition of the agonist, aggregation proceeded for 6 min and the area under the curve (A.U.C.; arbitrary units) of aggregation was calculated.

### 2.5 Statistical analyses

Paired samples *t*‐tests (IBM SPSS Statistics, v. 28, IBM Corp., Armonk, N.Y., USA) were used to compare T_c_, T_sk_, HR, MAP, RPP, FBF, and FVC between low‐dose ASA and placebo trials within subjects. Paired samples *t*‐tests were used to compare A.U.C. of aggregation between pre‐test placebo and pre‐test low‐dose ASA blood samples. Similarly, paired samples *t*‐tests were used to compare A.U.C. of aggregation between post‐test placebo and post‐test low‐dose ASA. A priori sample size calculation determined that 10 subjects would provide sufficient statistical power (>0.80, *α* = 0.05) to detect differences in T_c_ and skin blood flow responses related to ASA treatment. Given the underpowered sample size (*n* = 4) in the high‐dose ASA trials, these trials are exploratory only. Significance was accepted at *α* = 0.05. Data are presented as means ± SD.

## 3 RESULTS

**FIGURE 1:**
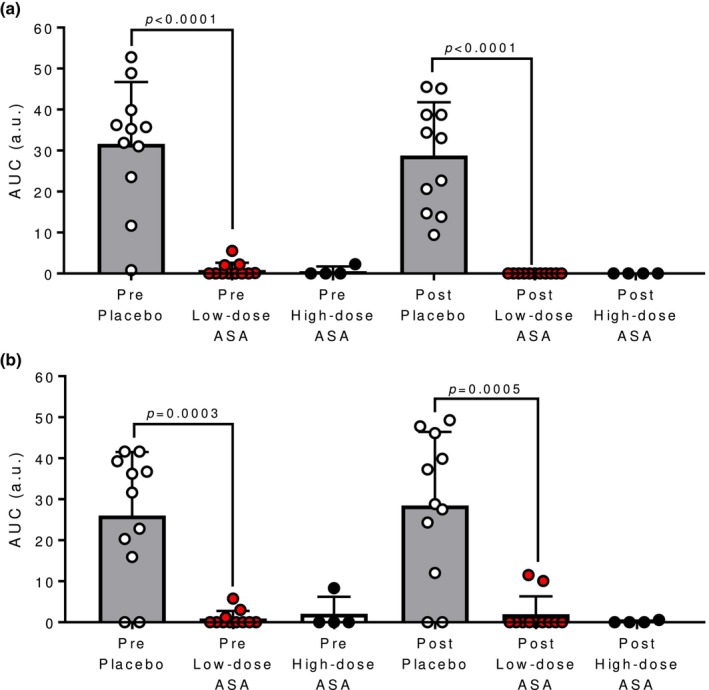
Effect of aspirin therapy on platelet aggregation measured by area under the curve (A.U.C.) of aggregation in response to 10 μL of 50 mM arachidonic acid in pre‐ and post‐test placebo (open circles), pre‐ and post‐test low‐dose aspirin (ASA; red circles) and pre‐ and post‐test high‐dose ASA blood samples in hot‐dry (A) and warm‐humid (B) environments.

As illustrated in Figure 1, ASA therapy significantly attenuated the A.U.C. of aggregation in response to AA pre‐ and post‐experiment in the hot‐dry (both *p* < 0.0001; Figure 1a) and warm‐humid condition (*p* = 0.0003 and *p* = 0.0005, respectively; Figure 1b).

**FIGURE 2:**
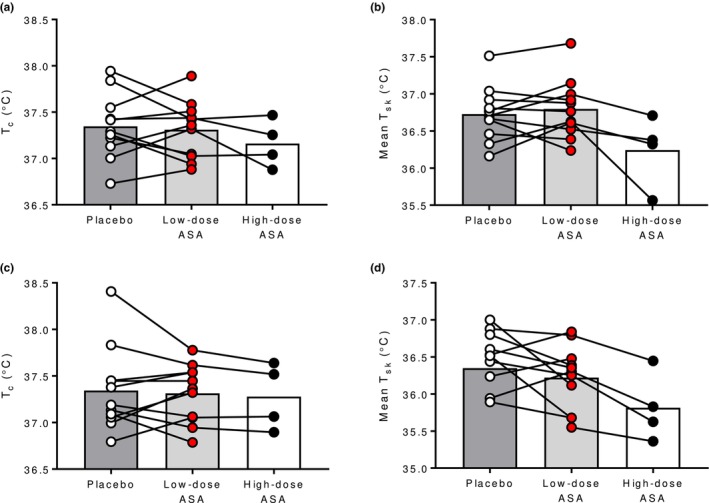
Core (T_c_) and mean skin temperature (${\bar{T}}_{\mathrm{sk}}$) in the hot‐dry (A and B) and warm‐humid environments (C and D) in placebo (open circles), low‐dose aspirin therapy (red circles), and high‐dose aspirin (closed circles) conditions. Each individual's response is illustrated by connecting lines.

**FIGURE 3:**
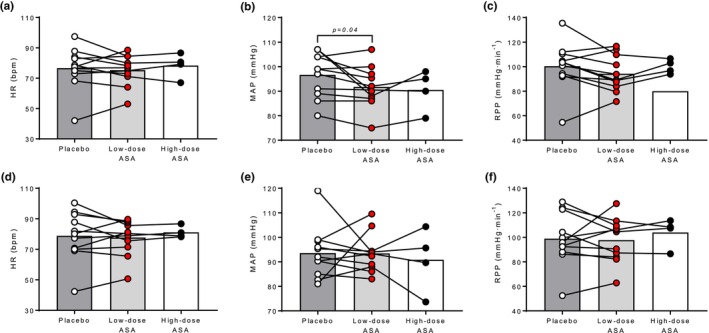
Heart rate (HR), mean arterial pressure (MAP), and rate pressure product (RPP) in the hot‐dry (A, B, and C) and warm‐humid environments (D, E, and F) in placebo (open circles), low‐dose aspirin therapy (red circles), and high‐dose aspirin (closed circles) conditions. Each individual's response is illustrated by connecting lines.

**FIGURE 4:**
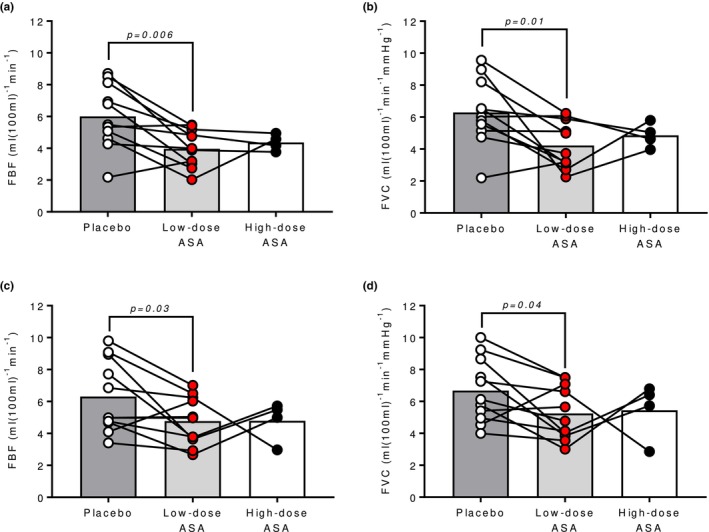
Forearm blood flow (FBF) and forearm vascular conductance (FVC) in the hot‐dry (A and B) and warm‐humid environments (C and D) in placebo (open circles), low‐dose aspirin therapy (red circles), and high‐dose aspirin (closed circles) conditions. Each individual's response is illustrated by connecting lines.

In both the hot‐dry and warm‐humid environment, the low‐dose ASA regimen had no effect on T_c_ or T_sk_ compared to placebo (all *p* ≥ 0.38; Figure 2). In the hot‐dry environment, MAP was lower in the low‐dose ASA trial compared to the placebo (*p* = 0.04; Figure 3b) but there were no differences in HR or RPP (*p* ≥ 0.13; Figure 3a,c, respectively). Similarly, in the warm‐humid condition, there were no differences in HR, MAP, or RPP between low‐dose ASA and placebo trials (all *p* ≥ 0.67; Figure 3d–f, respectively). In the hot‐dry environment, low‐dose ASA reduced FBF (*p* = 0.006; Figure 4a) and FVC (*p* = 0.01; Figure 4b). Similarly, in the WH environment, low‐dose ASA reduced FBF (*p* = 0.03; Figure 4c) and FVC (*p* = 0.04; Figure 4d). In the subset of participants that completed trials following a single 650 mg dose as an exploratory follow up, no differences were observed in either environment in any of the previously listed outcome variables compared to the low‐dose ASA trial or placebo trial (all *p* > 0.05).

## 4 DISCUSSION

The primary findings from the present study were that during minimal activities of daily living in older adults, (1) low‐dose ASA therapy inhibits platelet aggregation but does not affect core or skin temperature responses to heat stress in hot‐dry or warm‐humid environments and (2) low‐dose ASA therapy does attenuate skin blood flow in response to heat stress in the hot‐dry and warm‐humid environments. In the subset of participants that completed trials following a single maximum recommended 650 mg dose of ASA as an exploratory follow‐up to check for vascular COX inhibition, there were no effects on core or skin temperature or skin blood flow responses to heat stress. These data lend further support that platelet COX inhibition mediates the attenuated skin blood flow response. Altogether these data suggest that older adults who partake in low‐dose ASA therapy for primary prevention of cardiovascular disease are not at elevated risk of adverse heat‐related events during minimal activities of daily living under the conditions examined in this study.

Previously, our lab found that in middle‐aged adults, administration of low‐dose ASA therapy for at least 7 consecutive days resulted in attenuated reflex cutaneous vasodilation and more rapid elevations in T_c_ during passive whole‐body heating (Holowatz et al., 2010; Holowatz & Kenney, 2009). However, local vascular COX inhibition had no effect on cutaneous vascular conductance during hyperthermia (Holowatz et al., 2009, 2010), suggesting that platelet COX‐1 inhibition may have been responsible for the attenuated reflex vasodilation. Similarly, in the present study, skin blood flow was attenuated by a low‐dose ASA regimen in both hot‐dry and warm‐humid environments (Figure 4). There is evidence to suggest that inhibition of platelet COX‐1 alters the release of stimulating intraluminal factors that mediate downstream endothelium‐dependent events contributing to reflex vasodilation (Förstermann et al., 1988; McCord et al., 2006) and/or reduce shear‐mediated vasodilation (Green et al., 2010). In the present study, the efficacy of 7 days of low‐dose ASA to inhibit platelet COX‐1 was confirmed with impedance aggregometry (Figure 1). We recently validated the ability of impedance aggregometry to confirm the efficacy of ASA therapy to inhibit platelet COX (Williams et al., 2024) and found similarly here that platelet aggregation was eliminated in response to the agonist arachidonic acid.

While skin blood flow was attenuated in the ASA condition, there were no differences to core or skin temperature due to the low‐dose ASA therapy (Figure 2). This is contrary to previous findings that low‐dose ASA results in greater heat gain due to a faster rate of rise in core temperature during hyperthermia in middle‐aged adults (Bruning et al., 2013; Holowatz et al., 2010). We also previously demonstrated attenuated skin blood flow and altered thresholds for the onset of reflex cutaneous vasodilation for subjects exercising at moderate intensities in sustained hot environmental conditions (30°C, 40% relative humidity). However, compared to heating in water‐perfused suits or the prior higher‐intensity exercise protocol, the heat stress approach in the present study elicits an integrative physiological set of responses at a very low metabolic rate. The heat stress may not have been severe enough to elicit larger increases in core temperature sufficient to detect slight differences or attenuations that may have occurred due to the ASA treatment. Further, while the age range in the present study was 66–80 years, prior studies were conducted in primarily middle‐aged individuacls (50–65 years). Because of the differences in age and capacity of the thermoregulatory effector mechanisms (Holowatz et al., 2007; Kenney, 1988), the lack of effect on core temperature responses due to ASA may also be attributed to already attenuated thermoregulatory effector responses.

Although interpretation of results is limited due to the underpowered sample size, in the subset of participants that completed exploratory experimental trials following a single high dose (650 mg) of ASA, there were no differences in core or skin temperature or skin blood flow responses to heat stress in either environment. Given that a single high dose of ASA is sufficient to inhibit vascular COX (Patrono et al., 2001) and there were no differences in skin blood flow in the high‐dose trials, inhibition of vascular COX likely does not alter skin blood flow responses during hyperthermia. While the sample size is limited, these data suggest that attenuation in skin blood flow due to low‐dose ASA is a result of platelet COX‐1 inhibition rather than inhibition of vascular COX. This is consistent with previous data that demonstrate no changes in skin blood flow during hyperthermia when the local vascular COX inhibitor ketorolac was perfused directly into the skin microcirculation (Holowatz et al., 2009). Full systemic inhibition of platelet COX‐1 reportedly occurs within 3 days of low‐dose ASA treatment (Patrono et al., 1985, 2001). Therefore, the single 650 mg dose may not be sufficient to systemically inhibit platelet COX‐1 to alter skin blood flow responses given the subject ingested the ASA dose 1 h. prior to testing. However, platelet aggregation data from the present study suggest the high‐dose ASA inhibited platelet aggregation both pre‐ and post‐trial (Figure 1); therefore, the lack of attenuation in skin blood flow during high‐dose ASA trials may be due to the insufficient sample size to detect a difference. As a result of the insufficient sample size in the high‐dose ASA trials to detect a difference, further research is necessary to determine whether the single high‐dose regimen attenuates skin blood flow during heat stress.

Additionally, while all participants underwent a 2 h. exposure in both environmental conditions, outcome variables were only compared in a single environment that all participants experienced. Due to different starting temperatures and humidities between participants, a single common environment across all participants was chosen for comparison. Duration of exposure at the time when this environment occurred differed between participants due to different starting temperatures and humidities. However, within a participant, the environment used for comparison occurred at the same point in the 2‐h exposure for each condition as this is a within‐subject experimental design.

In conclusion, low‐dose ASA treatment in older adults resulted in attenuated skin blood flow responses but had no effect on core or skin temperature at low metabolic rates in either a hot‐dry or warm‐humid environment. While the mechanisms underlying the attenuation in skin blood flow remain speculative, these data and previous research suggest that platelet COX‐1 inhibition is the mechanism involved. Although skin blood flow was attenuated, low‐dose ASA therapy does not negatively impair thermoregulation in older adults. Therefore, these findings indicate that low‐dose ASA therapy does not increase the risk for adverse heat‐related events in older adults doing activities of daily living during environmental extremes.

## AUTHOR CONTRIBUTIONS

W.L.K. and L.M.A. conceived and designed research; K.G.F., R.M.C., and O.K.L. performed experiments; K.G.F. analyzed data; all authors interpreted results of experiments; K.G.F. prepared figures; K.G.F. drafted manuscript; all authors edited and revised manuscript; all authors approved final version of manuscript.

## FUNDING INFORMATION

This research was supported by the National Institute on Aging Grant T32 AG049676 to the Pennsylvania State University (R.M.C. and K.G.F.) and National Institutes of Health Grant R01 AG067471 (W.L.K.).

## CONFLICT OF INTEREST STATEMENT

The authors declare no conflicts of interest.

## ETHICS STATEMENT

All experimental procedures received approval from the Institutional Review Board at The Pennsylvania State University, and conformed to the guidelines set forth by the Declaration of Helsinki. After all aspects of the experimental procedures were explained, oral and written informed consent were obtained.

## Data Availability

Data will be made available upon reasonable request.
